# Hygiene

**Published:** 1858-07

**Authors:** 


Hygiene. We must not omit to notice a work of the past quarter on hygiene, by Dr. James H. Pickford.* It is an English systematic treatise on the subject. The book shows immense industry and great knowledge on the part of its author, and to sanitarians who require (and what sanitarian does not require it) a work of reference on sanitary science, this book is invaluable. To ourselves, as public teachers on Hygiene, we have felt practically the value of Dr. Pickford's labours, and have already availed ourselves of them for lecture-room purposes. For these reasons we recognize this book as of great worth, and recommend it as it deserves. But the construction of the book is, as we fear, opposed to that general success and usefulness which its author desires for it. It is full of facts, and is accurate in its details; but it is a book for the close student only. Unrelieved by the slightest touch of that artistic skill in literature which makes the new and difficult scientific lesson pleasurable, nay, bearable, it fails utterly as a genial instructor, and falls we fear from the press still-born. We hope Dr. Pickford will some day re-model his work. He has laid a foundation; let him now build an edifice. We describe his present work best by saying, that next to Oesterlen's "*Handbuch*", it is the most complete epitome of sanitary science extant.

We must this time be content to refer merely to Dr. M'William's able Report of the Commissioners of Her Majesty's Customs, and to his Statistical account of the health of the Water-guard and Water-side Officers; to the report of Dr. Edwin Snow (a distinguished American sanitarian) on the "Births, Deaths and Marriages of the City of Providence in 1857," and on "The History of Asiatic Cholera in Providence"; and to several admirable papers in the *Philanthropist* of the present year on "Prison Discipline and Diet," by Dr. Edward Smith.

---

* Hygiene. By James H. Pickford, M.D. London: Churchill. 1858.

